# Extra Supplement—The Nursing Mirror

**Published:** 1889-02-23

**Authors:** 


All Contributions for this Supplement should be addressed "The Nursing Mirror," care of The Editor, 38, Old Jewry, E.C.

### En Passant.

WORK IN THE WILDS.—Those nurses who were disappointed in obtaining the post of matron at Fiji, had better apply to Miss Lonsdale, of Manchester. The Rev. E. L. Wright has commissioned her to find a lady superintendent, with means of her own, to take charge of a hospital to be built at Lytton, British Columbia. The hospital is to be specially for the use of the Indians, who when they are ill, cry "Kismet!" (or its Indian equivalent), and lie down and die. The post will offer scope for quite new and original work, but it will be uncommonly hard work.

NATIONAL PENSION FUND FOR NURSES.—At a meeting of the council held on February 14th, Mr. Walter H. Burns in the chair, the honorary manager reported that the number of applications for pensions and sick pay up to date was 694 (exclusive of the Mildmay nurses and the nurses of the Seamen's Hospital, numbering about 133), of whom 540 had paid their contributions, amounting to £10,060, of which £1,130 had been received since the last monthly meeting. Fifty-seven applications had been received during the corresponding period, and 37 were accepted at the present meeting. It was reported that the funds invested amounted to upwards of £33,900. Only about 180 more nurses can now appear on the historic roll of the first thousand.

NURSES AS STEWARDESSES.—We wish to repeat once more the particulars which must be sent to the "Nursing Mirror" by those nurses who desire to become stewardesses. It is no use for us to approach the shipping companies on this question, till we have a sufficient number of suitable nurses on the register. Some very sensible and suitable women have expressed their desire to move in this matter, but others have sent in their names who are quite unsuitable. For instance, one nurse asks if she will dine with the first-class passengers if she goes as a stewardess! The following are the particulars to be given by would-be-stewardesses: (1) Name in full. (2) Age. (3) Where trained and what certificates held. (4) Previous occupation before becoming a nurse. (5) How many years engaged in nursing. (6) If a good sailor. (7) Single or widow. (8) Permanent address.

MISS BURFORD AT BOLTON.—The inhabitants of Bolton having decided to form a nursing association for that town, very wisely got Miss Burford, superintendent of the District Nurses' Association at Manchester, to explain to them how best to start about the work. Miss Burford made an excellent speech, during which she said a trained nurse must be a woman of inexhaustible patience, of good temper, and be able to work with method and judgment. Hospital nursing was comparatively easy. In the homes of the rich the nurse could have every assistance she required, but in the homes of the poor she had to work single-handed, to fight against disease and prejudice, the want of air, dirt, and drink. District nursing was not only helpful, but educational. In Manchester, the nurses attended from nine o'clock in the morning until one, and from half-past four until eight in the evening. Occasionally they went out in the afternoon to cases which were exceptionally bad. They attended every kind, with the exception of infectious diseases, and they took no case which had not been attended by a medical man, with the exception of dressing a wound or a burn. We believe the Earl of Bradford is to be president of the Bolton Association, and Mrs. Bridson honorary secretary. The annual subscriptions already promised reach £143, and the donations £200.

NEWS FROM PARIS.—Academical honours have lately been conferred on Mdlle. Bottard, for forty-eight years lady superintendent of the Hospice de la Salpetrière. The *Droit des Femmes* gives a biography of Madame Cahen, to whom the Cross of the Legion of Honour was presented on New Year's Day. Madame Coralie Cahen is the widow of a doctor, and when the Franco-German War broke out she was one of the first to join the Society for the Help of the Wounded. Throughout the whole campaign Madame Cahen worked unceasingly; she passed fearlessly through the German ranks when seeking the wounded, and she nursed with equal devotion the members of both armies. Yet Madame Cahen was thoroughly French, and would permit no insult to her beloved country. She had established an ambulance in the Lyceum at Vendome after the capitulation of Metz, and was caring for 800 wounded when the German officers hoisted their flag over the ambulance. Madame Cahen immediately threatened to depart with her staff and leave the 800 sick to their fate: she would never work except under the French flag she said. The German officers admired her courage and patriotism, and replaced the flag they had removed. When the war was over Madame Cahen travelled through Germany, seeking out her countrymen who were there detained in prisons or hospitals. The Empress Augusta so greatly admired this philanthropy that she presented Madame Cahen with the Red Cross of the German order. Madame Cahen is now described as a white-haired old lady with a gentle voice, who yet preserves much of her original strength of character.

THE LEEDS INSTITUTION.—The thirteenth annual meeting of the Leeds Trained Nurses' Institution was held on February 12th, when Mrs. E. Walker (the honorary secretary) read the following report: "The number of cases attended has been 789, and 114 cases have had to be declined owing to inability to supply a nurse at the time she was needed. The staff is now as under: Engaged in private nursing, 81; ditto, district nursing, 7; probationers in training at hospitals, 14; total, 102. Sixteen nurses completed their training during the year, and one probationer left at the conclusion of her training to nurse in her own home, paying the usual fine for her breach of agreement. The committee have to record with much regret the death of Nurse Noddings after a very brief illness. She was one of the senior nurses, and greatly valued for her conscientious and energetic work. There has been frequent and prolonged interruption to the work of the institution during the year in consequence of the illness of several of the nurses. One contracted typhoid fever whilst nursing a patient, and was off duty for 15 weeks; another was unable to nurse for 17 weeks in consequence of a severe strain from heavy lifting. Several other nurses have been off duty owing to ill-health for periods of from two to three months respectively. The committee would express their thanks to the medical officer of the institution for his constant care of the health of the nurses. The District Nursing Branch of the Institution is efficiently carried on; another district of the town has been taken up in response to an earnest appeal made at the last annual meeting. This makes the sixth division of the town now receiving the benefit of this charity. The committee commend this branch of work to the favourable notice of the public, as increased funds are still urgently needed to maintain its usefulness. A grant to it of £150 has this year been made from the surplus funds of the institution, but the deficiency at the close of the year amounted to over £30." The Chairman, in moving the adoption of the report, said that for three or four years past the institution, which he might call a society for mutual profit, being in no sense a charity, had had uninterrupted success, thanks in no little measure to the lady superintendent.

### Original Articles.

### POLITENESS.

A well-known physician was wont to say, "Politeness is a great friend to the doctors;" and the truth of this axiom is ever being more firmly impressed upon my mind. Sad, indeed, it is that the very graces of civilisation should thus turn against us, and that our endeavours to make ourselves pleasant, or at any rate not unpleasant, to our fellow-mortals should oftentimes result in our becoming exceedingly unpleasant to ourselves. Which of us have not experienced at some period of our lives the direful necessity of agreeing with feigned alacrity to a scheme which we know will inevitably land us in an attack of quinsy or a sick headache? And, unfortunately, the more softhearted you are, and the more considerate for other people's feelings, the worse it is for you. This is especially the case with young people who cannot bear to "make a fuss," and who, moreover, are, as a rule, more heavily weighted with polite scruples than their more hardened elders.

But, to set against this, the elders have some peculiar disadvantages to contend with. For instance, that terrible propensity of juvenile cooks and amateur culinary students to confer upon their seniors the honourable position of judges and tasters. What inward shudders assails one at the sound of, "Please do have some of this cake—I made it all myself;" r, "Now, uncle, you must let me help you. This *soufflet* is made on the very newest principles. You know I have had a term at South Kensington." There are modes—none of us can reach middle life without acquiring them—by which such delicacies as chocolate creams, pieces of toffee, bull's-eyes, etc., can be adroitly disposed of in the pocket or elsewhere without attracting the attention or wounding the feelings of infantile donors. But such elementary methods are out of the question in dealing with sharp-witted individuals in their teens or early twenties. Such determined benefactors are they that well-intentioned frauds are totally impracticable. Plates must be cleared, glasses and cups emptied, or the anxious and lynx-eyed preparers of the banquet, like the 20,000 Cornishmen, "will know the reason why."

The world has long been famed for knowing little of its greatest men and greatest deeds, and thus it comes to pass that small recognition is accorded to the acts of heroism constantly performed, under the circumstances sketched above, by long-suffering (suffering indeed!) fathers and mothers, uncles and aunts, grandparents, godparents, and family friends.

But these are not the only circumstances under which such torture may be inflicted, and heroically endured for politeness' sake. There are the occasions when one listens with quailings of secret foreboding to the genial voice of one's host, cheerily announcing, as he takes up carving knife and fork, "Now Mr. (or Mrs. or Miss) ——, you *must* let me give you a slice of this pork. I think you will say it is excellent. One of our own pigs; a peculiarly fine breed which I imported from ——." Of course, we are delighted to partake of the interesting dainty, and proceed forthwith to import for our own private nocturnal benefit a peculiarly fine breed of nightmares.

Or our gentle hostess kindly relates how she has had this cherry tart made expressly for us, recollecting our predilection for pastry. As a fact, we dislike cherries extremely, and she must evidently be confusing us with somebody else about the pastry, since it is an article from which we have long since learned to shrink with horror. But how hurt the kind creature's feelings? "Thank you very much. How kind of you!" and another devoted victim falls before the Juggernaut car of politeness.

But there are other occasions besides the somewhat obvious ones of meals which are utilised by this tyrannical benefactor of the medical art. Politeness takes us to a garden party on a damp, chilly day, and obliges us, when there, to sit with a counterfeit presentment of rural felicity in a draughty corner under a tree, while as for our feet (politely shod with soles of the consistency of paper), their "lodging is on the cold ground." Politeness further dictates the smiling acceptance of an ice, and the equally sprightly rejection of a wrap, which could easily be brought from the house by means of stopping one of the sets of tennis, and sending a player to tell the footman to direct the lady's maid to search for it.

Again, it is politeness which constrains us on a sultry July afternoon to sit for twenty minutes in a close, fusty drawing-room, blandly declining to have the windows opened, which "could be done of course in a moment if we really wish it," only, in our hostess's opinion, there would be considerable danger of draught. And oh! that awful item in politeness's programme—bugbear of my own youth—the necessity of maintaining an expression at least of intelligent interest, if not artistic ecstacy, while seated with one's back to the horses in a somewhat crowded carrriage on a hot summer day, being driven round to "see the beauties of the place." We are not ungrateful nor unappreciative; but the sun is blazing overhead; there is no room for our parasol; the "backwards" position produces direful effects upon our system; and all the while, it being a "personally-conducted tour," the eye of the forwards-sitting manager of the expedition is felt to be upon us—expectant or severe—and those facial muscles, which we are told are expressive of the emotions in animals, are strained to the utmost to repress undesirable signs of discomfort and to exhibit paroxysms of admiration and delight.

Let it not, however, be supposed that I would advocate a rude boorishness, or that I am not aware of the many diversified motives which may underlie an outward politeness—as self-interest, ambition, good nature, or real kindness of heart and unselfishness. Much more, of course, might be made of the subject, but in this unsubstantial chat I merely wish to expatiate a little, in surface fashion, upon the truth of the doctor's axiom which I have taken as my theme, and to offer, politely, some crumbs of sympathy to those of my polite readers who have found by sad experience how great a friend and patron of *ars medica* politeness is.

### Lectures.

An esteemed correspondent writes: There was quite a large gathering lately to hear a lecture on "The Matron." The lecturer's ideal was pretty much what might have been anticipated—a hospital version of the "perfect woman, nobly planned, to warn to comfort, to command"; but I must confess I thought she laid too much stress on the commanding. Even the medical staff were to transmit their orders to the nurses through her, and she was to have "absolute control of the female staff of the hospital." This, in face of the fact that the lecturer admitted, in reply to some remarks by Miss Homersham, that as yet women were not educated to fill posts of responsibility without losing their balance a little through the possession of absolute power, and that it was therefore well to have some court of appeal beyond the matron. But, as some one had pointed out, an appeal from the matron to the House Committee usually results in the matron's verdict being confirmed; so that such appeals are rather worse than a mere formality for the nurse or probationer. Miss Thompson also said a few words —though she allowed that they were not exactly relevant to the subject—about the custom of choosing sisters exclusively from the ranks of paying probationers, though the regular probationers were often as well suited for these posts, by reason of skill and natural refinement and gentleness, as the so-called lady probationers. She also spoke of private nurses, and advised that, in addition to their very moderate salaries, nurses should receive a percentage on their earnings, so that the popular ones might reap something besides fatigue and constant work from their popularity. Finally, the chairman said, alluding to a statement that probationers were mostly middle-aged women when they entered hospitals, that the profession of nursing seemed to have a remarkable power of turning back the hands of the clock of time, which sent us all away in a good temper with ourselves and our work.

### A Unique Wedding.

NURSES will marry sometimes, and, what is more, they occasionally marry doctors, much to the disgust of the prudes who are found in this as in every walk of life. But it is not often that a nurse pursues her vocation till the last moment, and then calmly walks to the altar in cap and apron. We hope some of our readers remember Miss A. A. Crisp who trained at the Queen's Hospital, Birmingham, and then passed on to Netley and Woolwich? Rather more than five years ago Miss Crisp emigrated to New Zealand to take charge of the nursing of the Auckland Hospital, and in October last Miss Crisp was married to Dr. Bond. The bride carried out her hospital duties to the very day or afternoon of her change of estate, and she retained to the last the costume of the profession which she has adorned. Miss Crisp was dressed in very fine silver-grey beige, with apron, collar, cuffs, and mob cap, with streamers to her cap, and carried a bouquet in her hand. She also wore on her left breast the medals received for service in South Africa and Egypt, besides one from the Turkish Government, while on her left shoulder rested the Royal Red Cross awarded by her Majesty the Queen. The bridesmaids were nineteen nurses all in uniform, and the crowd in the church was so great that the bride had some difficulty in forcing her way to the altar. Seldom before had a local function excited such interest in Auckland, and the crowd is scarcely to be wondered at when we consider how many of the inhabitants had had cause to thank either Miss Crisp or Dr. Bond for services rendered during their many years of public services. It was certainly a most picturesque and characteristic wedding, and if only the showers of good wishes which followed the happy couple could all be accomplished, their life would be a brilliant one. A few days before the wedding Miss Crisp was presented with an illuminated address, and a silver salver, silver-mounted cut crystal salad bowl (silver fork and spoon to match), and a chaste Crown Derby afternoon tea set. The presentation of the handsome gift was made by Mrs. Mackellar (wife of Dr. Mackellar) on behalf of the hospital staff. As the whole affair had been kept a secret from the recipient till the evening of the presentation, it came as a pleasant surprise. Dr. Bond responded for Miss Crisp, expressing her personal gratification at the unexpected gift, and that she would ever bear them all in happy remembrance. The silver salver bore the following engraved inscription: "Miss Crisp, Royal Red Cross, in loving remembrance from those who were associated with her in Auckland Hospital. 27th October, 1888."

### The Nurses' Bookshelf.

[Books for Review should be sent to The Editor, The Lodge, Porchester Square, W.

### Lectures for Nurses.*

WE have here a series of six surgical lectures delivered at Guernsey to the nurses of the cottage hospital. The lectures are based on an admirable system of commencing with an account of the processes of inflammation, and thence leading on to an account of abscesses, ulceration, gangrene, and the treatment of wounds. But what is worth doing at all is worth doing well, and the author should have paid more attention to grammar and punctuation: semicolons are conspicuous by their absence, and nearly every sentence has a weak ending. We quote the last paragraph of the book as a specimen of faulty construction. "So to repeat in treating wounds take care to give them rest and perfect cleanliness and freedom from the introduction of poisonous materials when attending to them."

*Lectures for Nurses. By E. L. Robinson, M.R.C.S.: Clarke, Guernsey.

### Everybody's Opinion.

*[Correspondence on all subjects is invited. No communications can be entertained if the name and address of the correspondent is not given, or unless one side of the paper only be written on.]*

### A LETTER FROM JAMAICA.

*(Concluded from page lxxix).*

I FELT rather disappointed to find no one to receive me on Kingston Quay. However, I made the best of it, got my goods and chattels through the Customs, and went to the Asylum. My announcement of myself as "Miss Blake, the new Matron," caused the utmost surprise, for the officials at Downing-street had never warned the Colonial authorities of my engagement even. The information, in point of fact, came in the same ship as myself, and this of course was the reason why there was no one to meet me. But that was not all! There had been no matron for more than a year, and my quarters were far from being ready. Nothing, however, could exceed the kindness shown me by the medical superintendent (Dr. Plaxton), his wife and sister. They received me in their own house for a week as their guest, and did all in their power to make me comfortable. Mrs. Plaxton, who had, to assist her husband, been acting as matron, spared no trouble to put me "through my paces," and gave me every information. This was of course of great use to me, for things are so different here that one has to learn almost the alphabet of it again. At first I did not suffer from *maladie du pays*; everything was so novel and interesting, and there was so much to learn and unlearn. Then I had to report myself to the Colonial Secretary, and the Chief of the Medical Department. But after I went to my own quarters I felt very lonely and homesick. I know that if I had had sufficient money with me for my passage, and had not signed a bond, I should have been home for Christmas! However, I have got over it now, and am beginning to look on the bright side of things. But even as recently as New Year's Eve, when our second doctor's wife kindly sent me a piece of real English holly with berries on it, which had come by steamer in ice, and a card conveying New Year's greetings, I quite broke down again. Kingston is a horrid town, I think; I never go there if I can possibly avoid it. Fancy the sewers running down the centres of the streets, and the kerb stones at some of the crossings a yard in depth! When you go shopping it is a case of "Hobson's choice": you take what you can get, not what you want, and pay dearly at that. The asylum is beautifully situated. On the one side the ocean comes into our grounds, and whichever other way we look we see the mountains and trees. I never tire of the sight and sound of the waves, though the latter is apt to make one melancholy. I never looked or felt so well in my life as I do now, which is not to be wondered at. "Early to bed, and early to rise," is our motto, and I am out of doors a great deal. I thoroughly enjoy my food, and sleep better than I did in London. Then from about 10 a.m. to 6 p.m. "the wind bloweth in from the sea;" when that drops, in the evening and all night we have the cool mountain breezes.

There is a great variety of fish, vegetables, and fruit to be had here; in fact, as the geography books say, "the best dessert in the world." It is a vegetarian's paradise, and I am sure much animal food is unnecessary. Things are very dear, too, except fruit. Mutton is 1s. and bacon 1s. 6d. per pound. Certainly beef and turtle are only 6d. Tea, sugar, and all imported goods are dear. I have very nice spacious quarters—drawing, dining, two bed-rooms, kitchen, stores, and bath-room, on one floor; a nice maid-servant (who is called a "butler" here) with an inmate to help her. She is a good cook, can make all kinds of nice little dishes suitable for a family of one; and as I cater for myself, of course if I do not like my food it is my own fault.

Housekeeping, as far as the staff is concerned, is characterised by a sweet simplicity. In fact there is none! I know all the matrons of institutions who read these lines will envy me when I tell them that our nurses and servants board, lodge, and wash for themselves. They get their meals in some mysterious fashion, when and how they can. If they are caught eating they look as though they were guilty of some great crime. Knowing as I do that the chief difficulty we have to contend with at home is trying to please the staff over their food, I know I have much to be thankful for. They work from 6 a.m. to 6 p.m. on day, and *vice versa* on night duty, and go home the rest of the time. Think of that! No mess-room or bed-rooms to look after! On the other hand, I have no assistance in supervision, and they want a deal of looking after. They cannot be trusted, as all are very unprincipled and untruthful, and take very little interest in their work. We have 225 female and 211 male patients, twenty-nine day nurses and servants, five night nurses. I don't know how many men are employed.

We have great difficulty in making the patients employ themselves, and the insanity is of a much lower type than among the white people: very little acute melancholia, comparatively few with decided delusions, and, amongst the women at any rate, very little either general or local paralysis. We have one woman who is not born yet, another who is dead, another whose head has been cut off by the doctor and another substituted for it, another who has lost her shadow for more than a year, several great ladies, and others who own the asylum, etc. Of course the majority are negroes, but some are half-castes, pure whites, or coolies. Some are appallingly ugly, but some are like lovely bronzes, splendidly formed, and with a good carriage, due to carrying weights on their heads, and to not wearing high-heeled boots.

On Christmas Day the patients had beef and plum-pudding for dinner, followed by sugar-cane and oranges, and a grand distribution of lucky-bags. The articles most prized were scented soaps, necklaces, ribbons, and small mirrors. It was very difficult to realise that it was the gay and festive season, for all that. We had had scarcely any rain, no fogs, frost, north-east winds, mud, slush, carols, waits, snow, or other joys (?) of Yuletide in England. No fires, or even fireplaces (we never have a fire the whole year round). The sun was shining as if it would never get tired of doing so. A couple of grass birds commenced building in the greenery round one of the dining-hall posts, under the delusion it was a new tree growing for their especial benefit. The trees were in blossom, the roses were blooming, everything in fact the very antithesis of any previous Christmas I had ever spent.

On Friday, December 28, the patients and nurses went to an entertainment in the theatre belonging to the asylum. This is a charming recreation hall. We have a very good stage, the best by far I have played on. We had an entertainment by a professor of the art of legerdemain, &c., first, followed by a farce, "Chiselling," in which friends of Dr. Plaxton took part. I played Mrs. Piper, a part I took almost the last time I was in any theatricals. Mrs. Plaxton had been indefatigable in superintending the fitting-up of the scenery, etc., and she and Miss Plaxton presided at the piano. All went off very well. The next night we had a very brilliant audience, composed of most of the beauty, rank, and fashion of Kingston and the neighbourhood, and including his Excellency the Governor, Lady Norman and suite. Evening dress was "the only wear." The officers from the barracks were in uniform. These theatricals were a great boon to me, as they afforded me occupation and recreation.

But my pen is running away with me. My mind—at any rate, my thoughts—are thousands of miles across the ocean. However, it is as good as the "Bad Boy's dere diry" to me to be able to write to you, dear Editor. I hope my friends in Birmingham, London, Exeter, and Macclesfield, to whom I have not yet had time to write, will accept this in lieu of a letter to them.—Yours truly, A. E. Blake.

### THE COMBINATION OF PRIVATE NURSES.

Handclasp writes:—In reply to your correspondent "F. F.," may I be allowed to make one or two suggestions as regards the ways and means of obtaining higher salaries for nurses? I. Could not the nurses belonging to public institutions and managed by committees—I am not including "Homes," so-called, generally got up by self-interested persons, and into which nurses should "look before they leap"—petition their several committees for increased remuneration, at the same time demanding, if refused, on what reasonable grounds they do so, nurses being quite amenable to reason and justice? II. In petitioning their committees, could not the nurses ask for a percentage on their earnings over and above their fixed salaries? III. Could not bonuses be granted, as is the case in some institutions I have heard of? IV. Would it not be possible to place balance-sheets before the nurses, and when all expenses were deducted from the incomings, the surplus, if any, be divided among the nurses according to their earnings, or otherwise, to be determined by the committees? I incline myself to a percentage on one's earnings. It is a foregone conclusion that the institutions would benefit by some such arrangement as I have attempted to describe, and possibly the training-schools would be induced to follow, for this reason—the majority of nurses would naturally go where they could earn most, and their places would be supplied by inferior women, or by those whose circumstances place them above any monetary considerations.

### NURSES AS STEWARDESSES.

Nurse Lang writes:—I should be very glad if you will place me on your list of sea-going nurses. I have been accustomed to the sea all my life, and have recently made the voyages from London to Brisbane, from Sydney to Calcutta, and from Bombay home. I previously served my training, for which I paid, at the General Hospital in this city. It was during the voyage from Bombay that I felt a knowledge of nursing should be one qualification in a stewardess. I brought a patient home, and the passengers were nearly all more or less invalided. I made myself acquainted with the stewardess' duties and applied at the P. and O. office for a berth. The reply was that the appointments were given to widows of men who had died in their service (this accounts for many women holding the office who are most unfitted for it), and the list was so full, that they could only go a voyage in their turn, thus being employed about four months in the year.

E.M.S. writes:—May I venture a few remarks on some of the unpleasant things we may expect to meet in going as nursing stewardness until people have learnt to see the good of it? Naturally there will be a good deal of jealously among the present class of stewardesses, and also the stewards, which will certainly be a trial to our tempers. I think no nurse should offer her services for a voyage without looking at it from every point of view, for it would be unfortunate to all parties did she discover her mistake at sea. But as I said before, I am willing to go a long voyage as assistant stewardess and on half pay, so as to be able to judge if nurses of culture and refinement would care to become a nursing stewardess, and so become a blessing to those sick at sea.

J. B. writes:—My friend sailed on one of the Inman Line steamers between New York and Liverpool. One journey she made to China, but she does not say by what line. She says in her letter to me, "My services were much appreciated by delicate lady passengers, and in one or two cases of hemorrhage from violent sea-sickness, my knowledge was very useful. There is no doubt that a nurse stewardess is quite essential in a long voyage, and the post can be made a very pleasant one. It was a pleasant change to me, after seven years of nursing." She was at sea three years.

### PRIVATE NURSES.

Nurse Rose writes.—It is evident what Institute is meant by your last week's correspondent, though she has taken such pains to give no clue to her own identity, which, considering the small amount of truth and justice in her remarks, is not surprising. During the last ten months I have been connected with the same Institute, and have received the utmost kindness and consideration in every way, especially during a fortnight's illness. Sister is always ready to meet the nurses' wishes in any reasonable way, and always wishes us to regard the Institute as home, doing everything she can to make it so. Until very lately there was no rule at all with regard to the Home, but on account of nurses meeting in the sitting room instead of taking rest and exercise, alterations were made. If a nurse does not bring home a good report, how can she expect a word of praise on returning from a case? but when she does deserve it I am sure she always gets it, and the house arrangements are most liberal. Those who will grumble must, but for my part I have seen no cause for complaint. I cannot vouch for the feelings of my fellow nurses on the point, not having ascertained them, but believe there are many who think as I do, that we are made as comfortable as it is possible.

### Appointments.

[It is requested that successful candidates will send a copy of their applications and testimonials, with date of election, to The Editor, The Lodge, Porchester Square, W.]

Bristol Royal Infirmary.—Miss M. Greenhough Smith, from Charing Cross Hospital, gold medalist of St. Bartholomew's, was elected, on February 15th, matron of the Royal Infirmary, Bristol.

Southend Victoria Hospital.—Miss Alice Richardson has been appointed nurse-matron to the above. She was trained at the London Hospital, and has for the past three and a-half years been engaged in district nursing in Poplar.

### HORRIBLE IGNORANCE.

A correspondent sends us the following cutting; it shows how necessary is the teaching of the St. John's Ambulance Association and other such societies:—"Dr. Iliffe, the Warwickshire coroner, held an inquest on Tuesday touching the death of Henry Williams (4), which took place on the previous Sunday night in consequence of burns. The mother of the deceased went out on an errand, leaving four children in the house, the eldest being a girl of eight. On returning, half an hour afterwards, she found the deceased had been burnt. There was no fender or guard in front of the fire. Questioned by the Coroner, she admitted that she generally left the children in this way, but that a neighbour occasionally looked in to see them. On her return on the day in question, she found two neighbours dressing the wounds with rags which had been soaked with urine. She did not think about going for a doctor, as the occurrence worried her very much, and she did not think of it. The neighbours told her they did not consider the injuries to be sufficiently serious to render a doctor necessary."

### Vacancies.

The following vacancies are announced:—

**Matrons.**

Teignmouth Infirmary; £35.

Bury Home for Incurables; £60.

Wrexham Infirmary; £40.

Hornsey Local Board, £30.

Jessop Hospital, Sheffield; £25.

Hornsey Infectious Hospital; £30.

Weymouth Royal Hospital, £35.

**Assistant Matron**

Holborn Union, £35.

**Nurses.**

Queen's Hospital, Birmingham; £20.

Truro Trained Nurses Home; £25.

Royal Hospital for Diseases of the Chest; £23.

Victoria Home, Bournemouth.

Swansea Nursing Institution.

Nottingham Nursing Institution; £25.

Nursing Institution, 96, 97, 98*a*, Wimpole Street, London, W., Mr. Wilson has several vacancies for thoroughly-trained Nurses.

St. John's Nurses Institution, Southport; several.

Royal United Hospital, Bath, Two (head); £26. Four (assistant) £12.

Eastbourne Nursing Institution; several.

Poplar and Stepney Sick Asylum; £21 10s.

Kingston Home (Hull).

Bath United Hospital (Two); £26.

Cumberland Infirmary (Night).

Holborn Union Infirmary; £17.

Eston Hospital; £25.

Southport Trained Nurses' Home.

Hospital for Epilepsy. Regent's Park.

Brockley Nursing Home.

Wakley Convalescent Home; £20.

Llandrindod Hospital; £16.

Bedford Nurses Institute.

South Devon Hospital, Plymouth, £25.

R. S. C. Hospital, Guildford. £16.

Hampstead Home Hospital and Nursing Institute; several.

Tewkesbury Hospital, £20.

London Temperance Hospital, Hampstead Road.

Essex and Colchester Hospital, £24.

**Probationers.**

Whitechapel Infirmary; several.

Tewkesbury Hospital.

Hammerwick Cottage Hospital, near Lichfield.

**Nurse-Housemaid.**

Sudbury Cottage Hospital, £15.

### Notes and Queries.

To Correspondents.—1. Questions may be written on post-cards. 2. Advertisements in disguise are inadmissible. 3. In answering a query please quote the number. 4. If a private answer is desired, a stamped addressed envelope must be forwarded.

**Queries.**

(110) *Midwifery Training.*—Will you kindly inform me where I can get free training in monthly nursing, and if there are any hospitals which give a small salary during training?—*Laura R.*

(111) *Register for Monthly Nurses.*—Is there a register through which a monthly nurse can obtain employment?—*Nurse S.*

(112) *African Nursing*—Where can I obtain particulars about nursing prospects at Cape Town?—*Banshee.*

**Answers.**

(113) *District Nurses.*—*Inquirer* is advised to apply to the Superintendent, National and Metropolitan Nursing Association, 23, Bloomsbury Square, W.C.

(114.) *Qualifications for a Nurse.*—Good health and an unselfish character are the chief qualifications. You must also be over 25 years of age. Apply to the matron at the nearest hospital.—*Experience.*

